# Mesenchymal Stem Cell Secretion of SDF-1α Modulates Endothelial Function in Dilated Cardiomyopathy

**DOI:** 10.3389/fphys.2019.01182

**Published:** 2019-09-24

**Authors:** Courtney Premer, Amarylis Wanschel, Valeria Porras, Wayne Balkan, Tatiana Legendre-Hyldig, Russell G. Saltzman, Chunming Dong, Ivonne Hernandez Schulman, Joshua M. Hare

**Affiliations:** ^1^Interdisciplinary Stem Cell Institute, Miller School of Medicine, University of Miami, Miami, FL, United States; ^2^Department of Medicine, Miller School of Medicine, University of Miami, Miami, FL, United States; ^3^Katz Family Division of Nephrology and Hypertension, Miller School of Medicine, University of Miami, Miami, FL, United States

**Keywords:** endothelial dysfunction, mesenchymal stem cells, allogeneic, dilated cardiomyopathy, SDF 1α

## Abstract

**Background:**

Endothelial dysfunction contributes to the pathophysiology of dilated cardiomyopathy (DCM). Allogeneic but not autologous mesenchymal stem cells (MSCs) improve endothelial function in DCM patients. We hypothesized that these effects are modulated by release of stromal derived factor-1α (SDF-1α).

**Methods:**

Plasma TNFα and endothelial progenitor cell-colony forming units (EPC-CFUs) were assessed at baseline and 3-months post-injection in a subset of POSEIDON-DCM patients that received autologous (*n* = 11) or allogeneic (*n* = 10) MSCs. SDF-1α secretion by MSCs, endothelial cell (EC) TNFα mRNA expression, and levels of reactive oxygen species (ROS) in response to SDF-1α were measured *in vitro*.

**Results:**

As previously shown, DCM patients (*n* = 21) had reduced EPC-CFUs at baseline (3 ± 3), which were restored to normal by allogeneic MSCs 3-months post-treatment (Δ10 ± 4). DCM patients had elevated baseline plasma TNFα (*n* = 15, 22 ± 9.4 pg/mL). Allogeneic MSCs (*n* = 8) decreased, and autologous MSCs (*n* = 7) increased, plasma TNFα (−7.1 ± 3.1 vs. 22.2 ± 17.1 pg/mL, respectively; *P* = 0.0005). In culture, autologous MSCs (*n* = 11) secreted higher levels of SDF-1α than allogeneic MSCs (*n* = 6) [76.0 (63.7, 100.9) vs. 22.8 (7.2, 43.5) pg/mL, *P* = 0.0002]. SDF-1α and plasma TNFα negatively correlated with EPC-CFUs in both treatment groups (*R* = −0.7, *P* = 0.0004). ECs treated with 20 ng SDF-1α expressed lower levels of TNFα mRNA than cells treated with 100 ng (0.7 ± 0.2 vs. 2.1 ± 0.3, *P* = 0.0008). SDF-1α at low but not high concentration inhibited the generation of ROS.

**Conclusion:**

MSC secretion of SDF-1α inversely correlates with EPC-CFU production in DCM patients and therefore may be a modulator of MSC therapeutic effect in this clinical setting.

**Clinical Trial Registration:**

https://clinicaltrials.gov/ct2/show/NCT01392625, identifier NCT01392625.

## Introduction

Dilated cardiomyopathy (DCM) is the most prevalent type of non-ischemic cardiomyopathy (Go et al., 2014). DCM has a multifactorial etiology, no specific therapies (Felker et al., 2000; Hare, 2010), and is accompanied by impaired endothelial function and increased markers of inflammation (Roura and Bayes-Genis, 2009). Endothelial dysfunction – here defined as diminished flow-mediated vasodilation (FMD) and endothelial progenitor cell (EPC) function – produces a cascade of detrimental events, including decreased nitric oxide (NO) bioavailability, inadequate EPC circulation and function, and increased oxidative/nitrosative damage and inflammation, which places further hemodynamic stress on the failing myocardium (Cai and Harrison, 2000; Munzel et al., 2008).

Administration of mesenchymal stem cells (MSCs) is a regenerative therapy approach under investigation for both DCM and endothelial function (Hare et al., 2009, 2017; Karantalis and Hare, 2015; Karantalis et al., 2015). MSCs are anti-inflammatory, antifibrotic, and pro-angiogenic, and thus, are of particular interest for treating endothelial dysfunction in patients with DCM (Williams and Hare, 2011). We previously demonstrated that DCM patients have endothelial dysfunction characterized by fewer endothelial progenitor cell-colony forming units (EPC-CFUs) and reduced FMD (Premer et al., 2015). Endothelial function improved significantly 3 months after transendocardial injection of allogeneic MSCs, but not in patients that received autologous MSCs (Premer et al., 2015). Patients receiving allogeneic MSCs also had greater improvements in New York Heart Association (NYHA) functional classification and reduced major acute cardiac events (MACE), and hospitalization rates compared to those receiving autologous MSCs (Mushtaq et al., 2014; Hare et al., 2017). While these results are encouraging and have significant implications for the treatment of DCM, the mechanisms underlying the benefits of allogeneic MSCs require further study.

Mesenchymal stem cells exert many of their therapeutic effects via the release of paracrine factors, including stromal derived factor-1α (SDF-1α), as well via the suppression of TNFα (Prockop and Youn Oh, 2012). We previously demonstrated that MSCs have an important role in cardiac stem cell migration, which is mediated through the SDF1/CXCR4 pathway (Hatzistergos Konstantinos et al., 2016). Additionally, it has been shown *in vitro* that there is an intimate relationship between TNFα and SDF-1α in cardiomyocyte mediated apoptosis (Jarrah et al., 2018). Accordingly, here we measured MSC secretion of SDF-1α and TNFα *in vitro* and plasma levels of TNFα in DCM patients that received MSC therapy to test the hypothesis that increased levels of SDF-1α impair the MSC-mediated improvement of endothelial function and reduction of plasma TNFα 3-months post-injection.

Our study shows that autologous MSCs in culture secrete higher levels of SDF-1α than do allogeneic MSCs. Importantly, patients that received autologous MSCs did not demonstrate improved endothelial function (Premer et al., 2015) or reduced plasma TNFα, 3-months after cell therapy. In contrast, patients that received allogeneic MSCs had significantly reduced plasma TNFα levels, accompanied by improved endothelial function 3-months after cell therapy (Premer et al., 2015). We further studied the connection between SDF-1α and oxidative/nitrosative stress *in vitro* and found that low concentrations of SDF-1α inhibit the generation of mitochondrial ROS, hydrogen peroxide, and nitrotyrosine by ECs. These results suggest that secretion of high levels of SDF-1α impair MSC efficacy, underlying the advantage allogeneic MSCs have relative to autologous cells for treatment of DCM.

## Materials and Methods

### Study Population

All patients were recruited from POSEIDON-DCM (NCT01392625), “A Phase I/II, Randomized Pilot Study of the Comparative Safety and Efficacy of Transendocardial Injection of Autologous MSCs vs. Allogeneic Mesenchymal Stem Cells in Patients” with non-ischemic DCM, and enrolled as part of a University of Miami sub-study entitled “Studying Endothelial Function and Endothelial Progenitor Stem Cells’ Colonies Before and After Heart Mesenchymal Stem Cell Transplantation” (endothelial trial #20110543). POSEIDON-DCM was a randomized clinical trial where patients received either 100 million autologous or allogeneic MSCs (Mushtaq et al., 2014; Hare et al., 2017). Autologous MSCs were harvested from the patient’s bone marrow via an iliac crest aspiration, 4–6 weeks before cardiac catheterization (Mushtaq et al., 2014; Hare et al., 2017). Allogeneic MSCs were harvested from the bone marrow of healthy donors via iliac crest aspiration (Mushtaq et al., 2014; Hare et al., 2017). All MSC products were manufactured by the University of Miami Cell Manufacturing Program, as previously described (Hare et al., 2012; Da Silva and Hare, 2013). MSCs were characterized based on the expression of CD105 and the absence of CD45 (Hare et al., 2012; Da Silva and Hare, 2013).

### Cell Culture

In order to measure MSC protein secretion, cryopreserved MSCs were rapidly thawed and plated on cell culture dishes in MSC medium (20% fetal bovine plasma, 1% penicillin/streptomycin, and MEM alpha). Cell culture media was changed every other day and upon reaching 70% confluence, MSCs were cultured with serum-free medium for 24-h and medium collected, spin at 1200 RPM, aliquoted, and frozen until assessed by ELISA. In POSEIDON-DCM, allogeneic MSCs were obtained from 6 donors. Cells from only 4 of those donors were used to treat the 10 patients in this study. However, all 6 donor MSC lines were analyzed for SDF-1α. Additionally, autologous MSCs from 11 patients were analyzed.

In order to analyze the effect of SDF-1α on ECs, human coronary artery endothelial cells (HCAECs) were grown to 70% confluency in tissue culture dishes. Cell culture media was changed every other day using EGM-2 BulletKit (Lonza CC-3162). HCAECs were then exposed to recombinant SDF-1α (Peprotech 300-28A). Additionally, cells were incubated with lipopolysaccharides (LPS; Sigma L4391) and subsequently treated with 20 ng of SDF-1α.

### Plasma Collection

Peripheral blood samples were obtained from each patient at baseline, and 3- and 6-months after MSC injection. Plasma was collected using a Ficoll-Paque density gradient and was subsequently frozen at –80°C until use.

### Enzyme-Linked Immunosorbent Assays (ELISAs)

Stromal derived factor-1α and TNFα from conditioned medium were measured according to the provided protocol. Blood samples were obtained from 15 patients, 8 who received allogeneic MSCs, and 7 who received autologous. TNFα was measured in patient plasma at baseline, and 3- and 6-months post injection according to the provided protocol. No samples were diluted to increase sensitivity of the assay.

### Endothelial Function Measurement

Endothelial progenitor cell-colony forming units were used to assess endothelial function and assays were performed as previously described (Premer et al., 2015). Briefly, EPC-CFUs were determined by isolating EPCs from peripheral blood samples, plating them on 6-well fibronectin-coated dishes followed by 24-well fibronectin-coated dishes in CFU-Hill medium (Stem Cell Technologies, cat #05900), and counting EPC-CFU formation on day five.

### Quantitative RT-PCR

Total RNA was isolated from HCAECs using RNAeasy mini kit (Qiagen). RNA (1 μg) was reverse-transcribed according to instructions utilizing a cDNA Synthesis kit (Applied Biosciences). Using Taqman Universal Master mix in an iQ5 real time PCR detection system (BioRad), qPCR was performed. All samples were run in duplicates and normalized to GAPDH Taqman gene-expression as follows: TNF1α: Hs00174128_m1; NOS2: Hs01075529_m1; and GAPDH: Hs02758991_g1. The change in mRNA expression was calculated compared to the change in normalized GAPDH, and values were expressed as fold change over the control.

### Immunofluorescence Staining and Fluorescence Assisted Cell Sorting (FACS) Analysis

Immunofluorescence staining was done on HCAECs cultured in EBM medium and plated onto LabTek CC^2^ chamber slides (Life Technologies). Various stimuli were applied to these cells: conditioned media from allogeneic MSCs, autologous MSCs and SDF-1α (20 ng/ml) (PeproTech). The cells were incubated for 1 h at 37°C and followed by stimulation with LPS (1 mg/ml) (Sigma-Aldrich) for 15 m at 37°C. These were then stained with MitoSox (Life Technologies) for 30 m at 37°C followed by DAPI (Sigma-Aldrich) for 10 m at 37°C. Specifically, cells were mounted onto glass slides with Mounting Medium Vectashield (Vector Laboratories). Images of cells were acquired using a Leica LSM 710 confocal microscope with 63× objective, followed by measurement of the average cell area of at least 10 cells per field using ImageJ software.

Additionally, HCAECs cells were stimulated with the treatments mentioned above and additionally with a positive control, TBHP (50 mM) (Invitrogen), and two samples treated with N-acetyl-cysteine (NAC, 250 mM) (Invitrogen) and LPS or TBHP, respectively. The cells were incubated for 3 h and further stimulated with NAC for 2 h and TBHP for 1 h, all at 37°C. These were then stained with DCF (Invitrogen) for 30 m and DAPI for 10 m at 37°C. As an additional measurement of oxidative stress, HCAECs were incubated with cellROX deep-red reagent and measured by fluorescence assisted cell sorting (FACS) (Bone et al., 2013). Staining for ROS was performed by incubating cells at 37°C with 5 μM CellROX Red reagent (C10491), following the manufacturer’s protocols (Invitrogen), and directly analyzed without fixing. Cell analysis was performed in FACS Canto-II (BD Biosciences). For MFI determination, we used the flow cytometry analysis software FlowJo. MFI refers to the fluorescence intensity of each event (on average) of the selected cell population, in the chosen fluorescence channel.

The cells stained for nitrotyrosine were stimulated with the previous stimuli and incubated at room temperature with 4% PFA for 10 min. The cells were blocked with 5% BSA (Sigma-Aldrich) for 1 h and then incubated with a primary antibody against nitrotyrosine (1:500, Sigma-Aldrich) overnight. The slides were then coated with the corresponding secondary antibody for 1 h. The cells were washed 3 times 5 min with PBS between every step and were further stained for DAPI for 10 m at 37°C.

### Statistical Analysis

To analyze the difference between autologous and allogeneic groups as well as to measure the difference before and after treatment in each group, an unpaired, two-tailed *t*-test was utilized. Correlations were measured using Pearson correlation, assuming a Gaussian distribution. Data are presented as mean and standard deviation of the mean. Both D’Agostino-Pearson omnibus normality test and Shapiro–Wilk normality tests were run to measure within-group variability on all data (only significant differences were reported as Mann-Whitney). Differences between groups regarding baseline characteristics were analyzed using a Fisher exact test. A one-way ANOVA was used for qPCR experiments. Lastly, immunofluorescence experiments were analyzed using a Student’s two-sided unpaired *t*-test with Welch’s correction for two group comparisons and a one-way ANOVA for multiple comparisons where *p* ≤ 0.05 was considered statistically significant.

## Results

### Baseline Characteristics

A total of 21 DCM patients were analyzed for this study, 10 received allogeneic MSCs and 11 received autologous MSCs (Table 1). The mean age of injected patients was 55.8 ± 11.2 years and 65% were male. There were no differences between groups regarding gender, race and ethnicity, patient history, baseline ejection fraction, left ventricular end diastolic function, six-minute walk test, peak VO_2_, or Minnesota Living with heart failure (HF). Lastly, TNFα plasma levels were similarly elevated at baseline in the two groups.

**TABLE 1:** Baseline characteristics of patients with dilated cardiomyopathy (*n* = 21).

**Baseline characteristics**	**Cell type**		***P*-value**
		
	**Allogeneic (*n* = 10)**	**Autologous (*n* = 11)**	
Age at injection (years)	58.3 (7.82)	55.909 (10.65)	0.99
Gender			
Male	7 (70%)	6 (54.5%)	0.5
Female	3 (30%)	5 (45.5%)	0.5
Ethnicity: Hispanic or Latino	1 (10%)	6 (54.5%)	0.03^∗^
Race: White	8 (80%)	5 (45.5%)	0.1
Race: Black	1 (10%)	0 (0%)	0.3
History of hypertension	4 (40%)	2 (18.2%)	0.3
New York heart association class			
Class I – No limitation	2 (20%)	4 (36.4%)	0.4
Class II – Slight limitation of physical activity	5 (50%)	6 (54.5%)	0.8
Class III – Marked limitation of physical activity	3 (30%)	1 (9.1%)	0.2
History of congestive heart failure	5 (50%)	5 (45.5%)	0.8
History of Valvular heart disease	1 (10%)	0 (0%)	0.3
History of smoking	7 (70%)	4 (36.4%)	0.3
History of diabetes	0 (0%)	1 (9.1%)	0.3
Peak VO_2_ (mL/kg/min)	17.23 ± 6.8	15.91 ± 5.94	0.6
Six minute walk test (meters)	437.78 m ± 66.95	425.27 m ± 74.66	0.7
Forced expiratory volume in one second (%)	2.43% (0.51)	2.41% (0.9)	0.9
MLHF: Median (IQR)	44.6 (24.75–68)	39 (21–67)	0.6
LV size and function			
Ejection fraction (%)	24.4% ± 5.52	21% ± 7.68	0.3
Left ventricular end diastolic volume (mL): Median (IQR)	253.8 mL (225.2–272.45)	193.2 mL (172.55–330.9)	0.7
Left ventricular systolic volume (mL): Median (IQR)	196.15 mL (171.73–219.95)	164 mL (125.5–290.8)	0.7
End diastolic diameter (cm): Median (IQR)	7 cm (6.63–7.18)	6.1 cm (5.85–7.65)	0.9
Biomarkers			
EPC-CFUs	3 ± 2 colonies	5 ± 3 colonies	0.08
TNFα	24.5 ± 5.6 pg/mL	25.3 ± 13.3 pg/mL	0.9

*Patient data are broken down by cell treatment, allogeneic (*n* = 10) vs. autologous (*n* = 11). Values are presented as *n*, *n* (%), or mean ± standard deviation. *T*-Tests and Chi-squared tests were used to compare groups, and statistically significant differences are indicated by bolding and an asterisk.*

### Only Allogeneic MSCs Reduced Elevated Baseline TNFα, Which Correlated With EPC-CFUs

**FIGURE 1:**
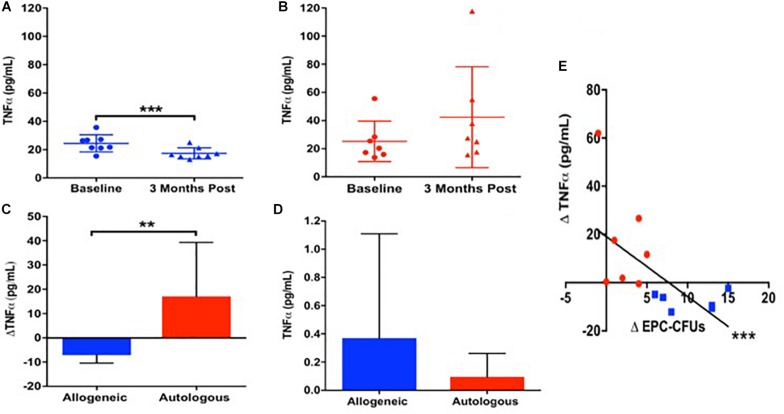
The relationship between patient TNFα and endothelial colony formation at baseline and 3-months post autologous and allogeneic mesenchymal stem cell (MSC) treatment. **(A)** Patients who received allogeneic MSC treatment (*n* = 8) had a significant reduction in plasma TNFα from baseline to 3 months post treatment (24.5 ± 5.6 to 17.4 ± 3.7 pg/mL, *P* = 0.0005^∗∗∗^). **(B)** Patients who received autologous MSC treatment (*n* = 7) had no change in plasma TNFα from baseline to 3 months post injection (25.3 ± 13.3 to 42.4 ± 33.1 pg/mL, *P* = NS). **(C)** There is a significant difference comparing the effect of allogeneic vs. autologous on the reduction of plasma TNFα 3 months post treatment (Δ−7.1 ± −1.9 vs. Δ17.1 ± 19.8 pg/mL, *P* = 0.009^∗∗^). **(D)** Neither allogeneic nor autologous MSCs are secreting notable levels of TNFα (0.37 ± 0.7 vs. 0.1 ± 0.2 pg/mL, *P* = NS). **(E)** There was a significant correlation between the change in plasma TNFα and the change in EPC colony formation from baseline to 3-months post injection (*n* = 15) (*R* = −0.7, *P* = 0.0004^∗∗∗^).

Given our previous findings that allogeneic MSCs improved endothelial function preferentially compared to autologous MSCs 3 months after administration (Premer et al., 2015), we investigated whether there was a differential inflammatory response since MSCs suppress inflammation (Prockop and Youn Oh, 2012), which in turn improves endothelial function (Shao et al., 2014). Accordingly, we measured plasma TNFα at baseline and 3-months after MSC infusion in patients with DCM. At baseline, patients (*n* = 15) had elevated levels of circulating TNFα (24.8 ± 10 pg/mL), which was similar between patients receiving allogeneic (*n* = 8) vs. autologous (*n* = 7) MSCs (24.5 ± 5.6 vs. 25.3 ± 13.3 pg/mL, *P* = NS, *T*-test). Three months after allogeneic MSC treatment, TNFα levels declined from 24.5 ± 5.6 to 17.4 ± 3.7 pg/mL (*P* = 0.0005; Figure 1A). In contrast, 3-months post-autologous MSCs treatment, TNFα levels were unchanged (25.3 ± 13.3 to 42.4 ± 33.1 pg/mL, *P* = NS; Figure 1B). There is a significant difference comparing the effect of allogeneic vs. autologous MSCs on the reduction of plasma TNFα 3 months post treatment (Δ−7.1 ± −1.9 vs. Δ17.1 ± 19.8 pg/mL, *P* = 0.009; Figure 1C).

Next, we examined TNFα release from cultured allogeneic and autologous MSCs, and found them to be similar (0.37 ± 0.7 vs. 0.1 ± 0.2 pg/mL, *P* = NS, *T*-test; Figure 1D). There was an inverse correlation between the change in plasma TNFα and the change in EPC colony formation from baseline to 3-months post injection (*n* = 15) (*R* = −0.7, *P* = 0.0004, Pearson Correlation; Figure 1E), highlighting that a positive change in EPC-CFUs post-treatment was associated with a reduction in plasma TNFα. These results suggest that the suppression of TNFα by allogeneic, but not autologous, MSCs plays a role in the mechanism mediating the beneficial effects of allogeneic MSC therapy in patients with DCM.

### SDF-1α Modulates Endothelial Function

**FIGURE 2:**
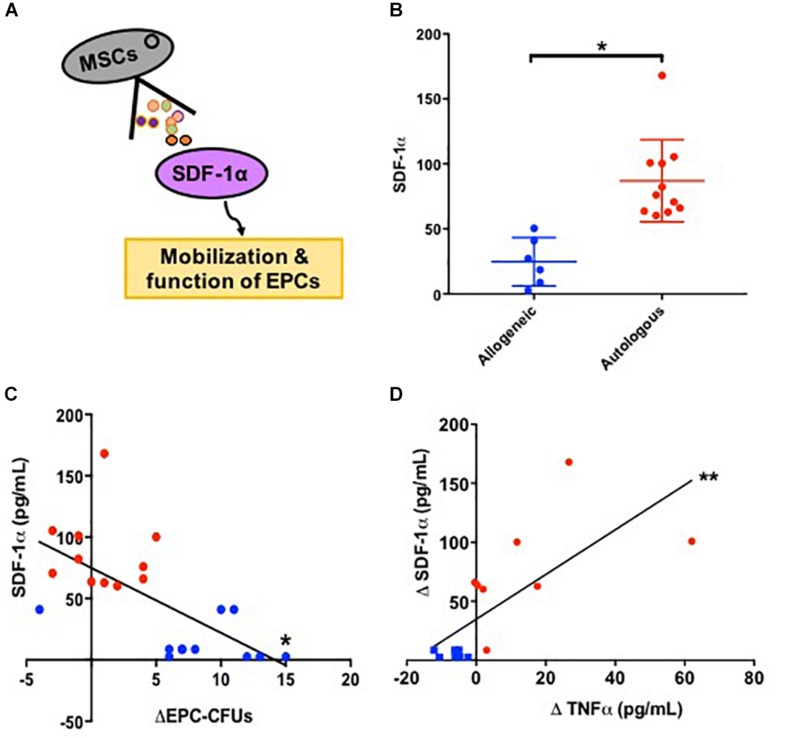
Stromal derived factor-1α (SDF-1α) as a biomarker for endothelial function. **(A)** Schematic illustrating that mesenchymal stem cells (MSCs) secrete SDF-1α which stimulates the mobilization and function of endothelial progenitor cells (EPCs). **(B)** Allogeneic MSCs (*n* = 6) secrete significantly lower levels of SDF-1α compared to autologous MSCs (*n* = 11) 22.8 (7.2, 43.5) vs. 76.0 (63.7, 100.9) pg/mL, *P* = 0.0005^∗^). **(C)** There was a significant inverse correlation between MSC SDF-1α secretion and the change in EPC-CFUs from baseline to 3-months post injection (*n* = 21, *R* = −0.7, *P* = 0.0004^∗^). **(D)** Additionally, there was a correlation between the amount of SDF-1α secretion from cultured MSCs and the change in TNFα from baseline to 3-months post injection compared to (*n* = 15) (*R* = 0.7, *P* = 0.003^∗∗^).

We next examined SDF1α levels, because this cytokine promotes mobilization of EPCs via the SDF-1/CXCR4 axis (Kijowski et al., 2001; Floege et al., 2009; Figure 2A). We measured SDF-1α release into serum-free culture medium by allogeneic and autologous MSCs. Autologous MSCs (*n* = 11) secreted 3.3 fold higher levels of SDF-1α compared to allogeneic MSCs (*n* = 6) [76.0 (63.7, 100.9) vs. 22.8 (7.2, 43.5) pg/mL, *P* = 0.0005, *T*-test; Figure 2B]. Subsequently, we examined the relationship between SDF-1α release *in vitro* and the change in EPC-CFUs *in vivo* from baseline to 3-months post MSC treatment and observed a significant inverse correlation between MSC SDF-1α secretion and the change in EPC-CFUs (*n* = 21, *R* = −0.7, *P* = 0.0004, Pearson Correlation; Figure 2C). Notably, patients receiving allogeneic MSCs had an increased number of EPC-CFUs post-treatment. Similarly, there was a correlation between the amount of SDF-1α secretion from cultured MSCs and the change in TNFα from baseline to 3-months post injection (*n* = 15) (*R* = 0.7, *P* = 0.003, Pearson Correlation; Figure 2D). These results suggest that high levels of SDF-1α impair MSC efficacy, negatively correlating to both EPC-CFUs and TNFα.

### SDF-1α Levels Modulate the Amount of Endothelial Cell TNFα Expression

**FIGURE 3:**
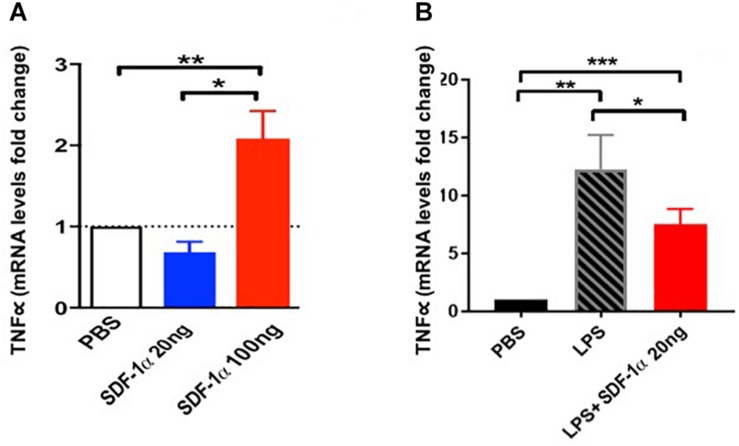
Stromal derived factor-1α levels modulate the expression of TNFα expression in human coronary artery endothelial cells (HCAECs). **(A)** HCAECs exposed to low levels (20 ng) of SDF-1α express low levels of TNFα mRNA (0.7 ± 0.2) comparable to the PBS control, whereas those exposed to high levels (100 ng) express high levels of TNFα mRNA compared to control (2.1 ± 0.3, *p* = 0.005^∗∗^). Furthermore, there is a significant difference comparing low dose vs. high dose (*p* = 0.004^∗^) as well as comparing all three groups together (*p* = 0.0008). **(B)** HCAECs stimulated with lipopolysaccharide (LPS) significantly upregulate TNFα mRNA (Δ11.3 ± 3, *p* = 0.001^∗∗^), and low dose (20 ng) of SDF-1α attenuate this elevation (Δ−4.8 ± 1.6, *p* = 0.03^∗^). ^∗^*p* < 0.001.

We further tested the link between SDF-1α, TNFα, and ECs *in vitro*. Human coronary artery endothelial cells (HCAECs) exposed to low levels of recombinant SDF-1α (20 ng) expressed low levels of TNFα mRNA (0.7 ± 0.2), whereas HCAECs exposed to high levels of recombinant SDF-1α (100 ng) expressed high levels of TNFα (2.1 ± 0.3, *p* = 0.0008 between groups, ANOVA; Figure 3A). Furthermore, cells treated with 100 ng SDF-1α produced significantly higher levels of TNFα compared to cells treated with PBS, whereas cells treated with 20 ng SDF-1α had similar TNFα levels as PBS-treated cells. This result suggests that high levels of SDF-1α increase TNFα mRNA in ECs.

We next tested if low levels of SDF-1α reduce TNFα upregulation in response to LPS *in vitro.* We incubated HCAECs with LPS to induce an immune response and subsequently treated cells with 20 ng SDF-1α. There was a significant increase in TNFα mRNA after treatment with LPS (Δ11.3 ± 3, *p* = 0.001, ANOVA), and 20 ng SDF-1α counteracted this increase (Δ−4.8 ± 1.6, *p* = 0.03, ANOVA; Figure 3B). These results demonstrate that SDF-1α modulates the expression of TNFα in ECs *in vitro.* Notably, these results highlight the observed allogeneic over autologous advantage, where allogeneic MSCs – which produce low levels of SDF-1α – and autologous MSCs – which produce high levels of SDF-1α – differentially effect TNFα in patients 3 months post treatment.

### SDF-1α Is Protective Against Both Mitochondrial Reactive Oxygen Species (ROS) and Nitrosative Stress

Oxidative stress initiates a wide array of deleterious processes that contribute to endothelial dysfunction. Therefore, we investigated whether SDF-1α and MSC conditioned medium, which contains SDF-1α, could prevent ROS production in ECs.

**FIGURE 4:**
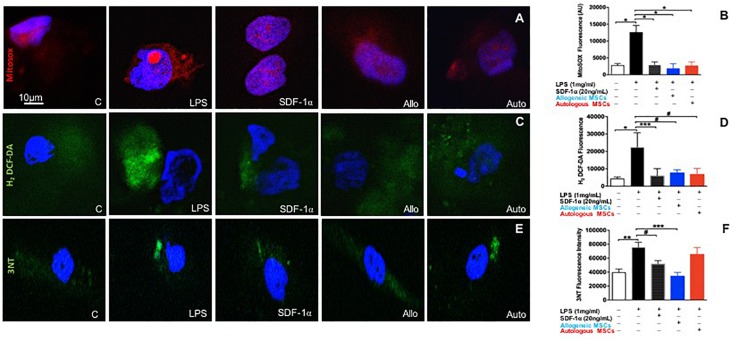
Low dose SDF-1α is protective against both mitochondrial reactive oxygen species (ROS) AND nitrosative stress. **(A)** Representative immunofluorescence images of human coronary artery endothelial cells (HCAECs) incubated with the mitochondrial O_2_^–^ – sensitive fluorescent dye MitoSOX Red (10 um), and DAPI (blue) after treatment with SDF-1α recombinant (20 ng; *n* = 4), allogeneic MSC conditioned medium (*n* = 4) or autologous MSC conditioned medium (*n* = 5) and stimulated with LPS (1 ug/mL). **(B)** Quantification of MitoSOX Red fluorescence showed that LPS significantly increased superoxide production compared to control (301676 ± 7843 vs. 112664 ± 22307, *p* < 0.0001^∗^), whereas SDF-1α, allogeneic MSC conditioned medium, and autologous MSC conditioned medium prevented superoxide production induced by LPS (58674 ± 8145, 52665 ± 33625, 39535 ± 13687 AU, *p* < 0.0001^∗^, respectively. **(C)** Representative immunofluorescence images via confocal microscopy of HCAECs incubated with H_2_-DCF-DA (Green) that detects intracellular peroxides including H_2_O_2_ and DAPI (blue). **(D)** Quantification of the average of DCF fluorescence showing LPS induced significantly higher rates of hydrogen peroxide production compared to control (22491 ± 16371 vs. 4464 ± 2047, *p* < 0.0001^∗^), while SDF-1α, allogeneic MSC conditioned medium, and autologous conditioned medium attenuated cell hydrogen peroxide production (6172 ± 3920 AU, *p* < 0.01^∗∗∗^, 9753 ± 4751 AU, *p* < 0.05^#^, 46941 ± 89798, *p* < 0.05^#^, respectively). **(E)** Representative pictures of nitrotyrosine (3NT) expression in HCAECs stimulated with LPS followed by SDF-1α, allogeneic MSC conditioned medium, and autologous conditioned medium. **(F)** Quantification of 3NT fluorescence demonstrating that LPS significantly increased nitrotyrosine expression (75894 ± 13874 vs. 43546 ± 10020 AU, *p* < 0.001^∗∗^, and only SDF-1α and allogeneic MSC conditioned medium reduced nitrotyrosine expression (52132 ± 4395 AU, *p* < 0.05^#^, 34761 ± 4898 AU, *p* < 0.0001^∗∗∗^). Autologous MSC conditioned medium did not block LPS induced nitrotyrosine expression (56949 ± 21648 vs. 75894 ± 13874, respectively, *p* = NS).

Human coronary artery endothelial cells were pre-treated with either low dose recombinant SDF-1α (*n* = 4), or allogeneic MSC- (*n* = 4), or autologous MSC-conditioned medium (*n* = 5), then stimulated with LPS for 6 h, and subsequently incubated with cell permeable probes sensitive to superoxide (MitoSOX Red) or hydrogen peroxide (H_2_O_2_; H2-DCF-DA). MitoSox red, a mitochondrial superoxide indicator, was used to label specifically mitochondrial superoxide followed by DAPI (nuclei dye) in HCAECs. Images were captured by confocal microscopy. As expected, LPS significantly increased superoxide production compared to control (301676 ± 7843 vs. 112664 ± 22307, *p* < 0.0001, ANOVA; Figures 4A,B). Low dose SDF-1α, allogeneic MSC conditioned medium, and autologous MSC conditioned medium prevented superoxide production induced by LPS (58674 ± 8145, 52665 ± 33625, 39535 ± 13687 AU, *p* < 0.0001, respectively, ANOVA; Figures 4A,B).

We next looked at H_2_O_2_ production rates using DCF-DA, and again found that LPS induced markedly higher rates of H_2_O_2_ production compared to control (22491 ± 16371 vs. 4464 ± 2047, *p* < 0.0001, ANOVA), while low dose SDF-1α, allogeneic MSC conditioned medium, and autologous MSC conditioned medium attenuated cell H_2_O_2_ production (6172 ± 3920 AU, *p* < 0.01, 9753 ± 4751 AU, *p* < 0.05, 46941 ± 89798, *p* < 0.05, respectively, ANOVA; Figures 4C,D).

To lend further confidence to our results, we measured ROS by an alternative method, flow cytometry (Supplementary Figure S1A). HCAECs were incubated with cellROX deep-reagent and measured by FACS. We observed that HCAECs pre-treated with low dose SDF-1α, allogeneic MSC conditioned media, or autologous MSC conditioned media lowered TBHP-induced ROS production by 50, 33, and 30%, respectively. These results are consistent with those observed above.

Next, we measured the effect of SDF-1α and MSC conditioned medium on nitrotyrosine levels, a biomarker of reactive nitrogen species formation and an indicator of protein oxidative damage (Figures 4E,F). LPS significantly increased nitrotyrosine expression (75894 ± 13874 vs. 43546 ± 10020 AU, *p* < 0.001, ANOVA; Figures 4E,F). Interestingly, only low dose SDF-1α and allogeneic MSC conditioned medium decreased nitrotyrosine expression (52132 ± 4395 AU, *p* < 0.05, 34761 ± 4898 AU, *p* < 0.0001, respectively, ANOVA; Figures 4E,F). Autologous MSC conditioned medium did not block LPS induced nitrotyrosine expression (56949 ± 21648 vs. 75894 ± 13874, respectively, *p* = NS; Figures 4E,F).

In order to further validate our nitrotyrosine experiment, we checked the mRNA levels of iNOS (NOS2). Pathologic nitric oxide production by iNOS can be the source of nitrosative stress. We performed a curve dose response to SDF-1α in HCAECs stimulated with LPS. Low amounts of SDF1α were able to downregulate the increased mRNA levels of NOS2 stimulated by LPS treatment (4.07 ± 0.03 vs. 9.85 ± 1.62, *p* < 0.01, ANOVA), however, higher amounts of SDF1α did not change NOS2 mRNA levels (Supplementary Figure S1B). Together, these findings demonstrate that low (physiological) amounts of SDF-1α may serve a protective role against ROS and nitrosative stress, which mechanistically underlie endothelial dysfunction.

## Discussion

**FIGURE 5:**
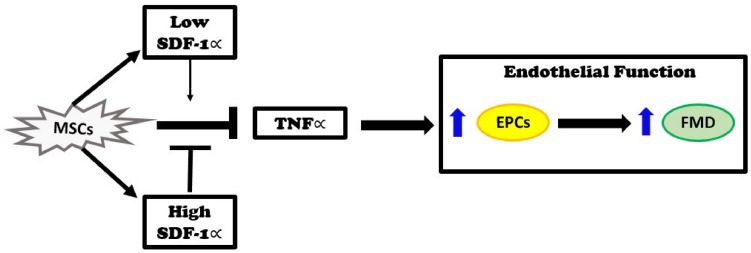
Injection of MSCs into patients with dilated cardiomyopathy reduces elevated TNFα levels and improves endothelial function as measured by an improvement in endothelial progenitor cell (EPC) colonies and flow-mediated vasodilation (FMD). These effects remain intact when SDF-1∝ levels are low. On the other hand, when SDF-1α levels are high, the beneficial effects of MSCs are inhibited.

The major new findings of this study are that autologous MSCs secrete higher levels of SDF-1α *in vitro* than allogeneic MSCs, and that these levels inversely correlate with EPC bioactivity in DCM patients. Three months after infusion, DCM patients that received autologous MSCs had no improvement in plasma TNFα levels. In contrast, patients that received allogeneic MSCs had reduced TNFα plasma levels. Furthermore, *in vitro*, SDF-1 α at low concentration offset mitochondrial ROS and nitrosative stress. Based on our previous observations that allogeneic, but not autologous, MSCs improve endothelial function as measured by EPC-CFU formation and FMD% (Mushtaq et al., 2014; Premer et al., 2015; Hare et al., 2017), together with the findings presented here, we propose that the elevated levels of SDF-1α inhibit the beneficial effects of MSCs. Allogeneic MSCs obtained from young, healthy donors secreted lower levels of SDF-1α *in vitro*, which correlated with reduction of TNFα and improved endothelial function, as indexed by increasing EPC-CFUs and FMD, in DCM patients (Figure 5).

Patients with DCM present with endothelial dysfunction as measured by reduced EPC-CFUs and impaired FMD. Many clinical trials have established the beneficial effects of MSCs in HF (Hare et al., 2009, 2012, 2017; Mushtaq et al., 2014; Karantalis and Hare, 2015; Kanelidis et al., 2016), due at least in part to paracrine effects. However, there is a paucity of studies identifying the specific factors secreted by MSCs responsible for these effects, as well as few studies addressing the DCM patient population. *In vitro* studies show that MSCs secrete anti-inflammatory factors and cytokines, such as IL-2, TGF-β1, hepatocyte growth factor, nitric oxide (NO), prostaglandin 2, and SDF-1α, that can modulate the mobilization of EPCs from bone marrow (Iyer and Rojas, 2008; Karantalis and Hare, 2015). Furthermore, MSCs stimulate angiogenesis and vasculogenesis, as well as suppress inflammation (Williams and Hare, 2011). However, no studies have identified the mechanisms underlying MSC efficacy and demonstrated the effect on endothelial function in a clinical model. Here we examined MSCs from patients enrolled in the POSEIDON-DCM clinical trial, along with various circulating factors and parameters of endothelial function, to gain novel insights into the differential effect of autologous vs. allogeneic MSCs on endothelial function. We previously showed that patients in the POSEIDON-DCM clinical trial who received allogeneic MSCs had improved endothelial function, whereas patients who received autologous MSCs did not (Premer et al., 2015). Furthermore, we showed that only cell culture medium from allogeneic MSCs was able to restore endothelial tube networks that were impaired by treatment with the nitric oxide inhibitor, L-NG-Nitroarginine methyl ester N omega-Nitro-L-arginine methyl ester hydrochloride (L-NAME)^5^. These findings led us to explore the role of SDF-1α in mediating these effects due to its previously described role in stimulating EPC function, EPC migration, and vasculogenesis (Petit et al., 2007). Notably, here we demonstrated an important and novel cytokine that could explain, at least in part, the differences between autologous and allogeneic MSC therapy in these patients. We showed that allogeneic MSCs secrete significantly lower levels of SDF-1α than autologous MSCs *in vitro*, which was associated with reduced systemic TNFα and increased EPC bioactivity in patients, thereby improving arterial physiologic vasodilatory response and decreasing unfavorable cytokine mobilization, 3-months post injection.

Delineating the interplay between SDF-1α and TNFα is an emerging area of research regarding EC function. Classically, SDF-1α signaling has positive effects on stem cell homing, stimulating EPC circulation, and enhancing neovascularization (Askari et al., 2003; Wojakowski et al., 2008). However, we found a paradoxical effect in which very high levels of SDF-1α secretion by autologous MSCs *in vitro* was associated with an increase in the already elevated levels of TNFα in DCM patients, ultimately offsetting positive effects on endothelial function. In agreement with this concept, Han *et al.* showed that SDF-1α activates NF-kB, which in turn stimulates TNFα production, inducing cell death in primary astrocytes (Han et al., 2001). Similarly, it was recently reported that high doses of SDF-1α induce TNFα-mediated apoptosis in adult rat cardiomyocytes and that this effect is dose dependent, with lower doses appearing to be protective against basal cell death (Jarrah et al., 2018). Shibata et al. (2013) also illustrated the negative effects of SDF-1α overexpression, which results in gastric dysplasia in which inflammation is greatly accelerated. Ultimately, these studies are consistent with our MSC findings, in which autologous MSCs secreted elevated levels of SDF-1α resulting in detrimental effects on inflammation as measured by plasma concentrations of TNFα.

Furthermore, TNFα contributes significantly to pathologic inflammation and vascular dysfunction (Feldman et al., 2000; Zhang et al., 2009) and is elevated in patients with DCM (Feldman et al., 2000; Ridker et al., 2000). However, our study is the first to demonstrate that MSCs isolated from healthy donors reduced these circulating pathologic levels 3 months post injection, while MSCs isolated from patients with DCM had no effect on reducing TNFα levels. In our recent report of the primary findings of POSEIDON-DCM (Hare et al., 2017), we examined TNFα levels 6 months post allogeneic and autologous MSC administration. Interestingly, we found that both treatments reduced elevated TNFα, however, allogeneic MSCs were twice as effective at reducing these levels. This result suggests that there is an attenuated response to autologous MSC cell therapy, at 3 months there is no reduction, while at 6 months there is a small reduction (Hare et al., 2017). Tögel et al. (2005) illustrated that patients with kidney disease who received MSCs were protected against ischemic renal failure via a paracrine mechanism mediated by reducing TNFα, IL-1β, and IFN-γ^33^, paralleling our results in DCM patients. In addition, we have shown in an elderly frail population that intravenous infusion of allogeneic MSCs decreases TNFα plasma levels and improves physical performance measures and quality of life (Golpanian et al., 2017; Tompkins et al., 2017). Moreover, in the current study, TNFα levels correlated with SDF-1α and EPC-CFUs, providing mechanistic insight. In line with these findings, Hong et al. (2015) found that TNFα down-regulated EPC expression resulting in reduced numbers of EPC-CFUs as well as diminished incorporation into HUVEC networks in children with vasculitis.

There are several limitations to our study, which is part of a clinical trial. Cytokine experiments were done *in vitro*, and the association between SDF-1α, TNFα, and EPC-CFUs was based on robust correlations combined with the known signaling pathway for SDF-1α (Kijowski et al., 2001). Notably, our *in vitro* EC experiments in which recombinant SDF-1α modulated the expression of TNFα lend confidence to our conclusions. Furthermore, previous studies support our findings. Specifically, the role of SDF-1α in reducing oxidative stress *in vivo* has been demonstrated in a number of murine studies. Blocking the SDF-1α receptor with ADM3100 in mice receiving LPS resulted in increased oxidative stress in a variety of organs, as compared to mice receiving LPS alone (Seemann and Lupp, 2016). Indeed, administration of an SDF-1α analog (CTCE-0214D) showed antioxidative properties by increasing the expression and activity of heme oxygenase 1 (HO-1), an antioxidant enzyme (Seemann and Lupp, 2016). In addition, it has been shown that high levels of ROS lead to EC dysfunction and death (Irani, 2000; Panth et al., 2016) by damaging macromolecules, decreasing NO bioavailability (Godo and Shimokawa, 2017), and inhibiting release of endothelium-derived hyperpolarizing factors (EDHF) (Eizawa et al., 1995; Godo and Shimokawa, 2017). Zhang et al. (2016) reported that SDF-1α has a direct rescue effect on oxidative stress limiting ROS generation at the mitochondrial level. Dinić et al. (2016) showed that SDF-1α protects pancreatic β-cells from the toxic effects of H_2_O_2_ by enhancing CAT activity and expression. Another limitation to our study is that a cytokine panel was run on 3 patients in order to choose cytokines on which to focus. Future studies will aim to delineate the difference between allogeneic and autologous MSCs through large-scale secretome analysis. Moreover, the number of patients in this study was relatively small, while there were 21 patient samples for EPC-CFUs and SDF-1α analysis, only 15 of those patients had blood samples available for TNFα analysis. Nonetheless, the differences between allogeneic and autologous MSC treatment outcomes were striking, lending confidence to the results. Patients who received autologous MSCs had a bone marrow aspiration prior to injection, while patients who received allogeneic MSCs did not. Although this extra intervention may have caused a minor acute inflammatory response, patients did not receive cells until 4–6 weeks after the aspiration to ensure recovery; therefore, it should have little to no impact on the results. Lastly, we were not able to investigate whether cells delivered to the heart can be found in the circulation of these patients.

Ultimately, this study demonstrates a potent and clinically relevant efficacy outcome of transendocardial injection of MSCs in patients with DCM. Allogeneic MSCs secreted lower levels of SDF-1α *in vitro* than autologous MSCs, which correlated with changes in plasma TNFα levels in DCM patients treated with MSCs. As impairment in the endothelial function of patients with cardiovascular disease is known to be highly predictive of adverse outcomes and disease progression, targeting endothelial function represents an important therapeutic strategy. Together, these findings reveal a mechanism mediating the effects of MSCs on endothelial function and have important implications for the use of allogeneic and autologous MSCs in patients with DCM.

## Data Availability

All datasets for this study are included in the manuscript and the Supplementary Files.

## Ethics Statement

The studies involving human participants were reviewed and approved by the University of Miami, IRB. The patients/participants provided their written informed consent to participate in this study.

## Author Contributions

CP, IS, and JH contributed to the conception and design of the study. CP, VP, AW, TL-H, and RS performed the experiments and data collection. CP and WB performed the statistical analysis. CP wrote the first draft of the manuscript. IS, WB, CD, and JH wrote the sections of the manuscript. All authors contributed to the manuscript revision, read, and approved the submitted version.

## Conflict of Interest Statement

CP and JH reported having a materials and methods patent for endothelial dysfunction. JH reported having a patent for cardiac cell-based therapy. He holds equity in Vestion Inc., and maintains a professional relationship with Vestion Inc., as a consultant and member of the Board of Directors and Scientific Advisory Board. JH is the Chief Scientific Officer, a compensated consultant and advisory board member for Longeveron, and holds equity in Longeveron. JH is also the co-inventor of intellectual property licensed to Longeveron. Longeveron, LLC and Vestion Inc., did not participate in funding this work. The remaining authors declare that the research was conducted in the absence of any commercial or financial relationships that could be construed as a potential conflict of interest.

## Supplementary Material

FIGURE S1 **(A)** HCAECs were incubated with cellROX and expression of ROS was measured via FACs. HCAECs pre-treated with SDF-1α allogeneic conditioned media and autologous conditioned medium lowered ROS production stimulated by TBHP in 50, 33, and 30%, respectively. **(B)** qPCR analysis of HCAECs stimulated with lipopolysaccharide (LPS) showed significantly upregulated NOS2 mRNA levels (9.85 ± 1.62 vs. PBS control 0.99 ± 0.01, *p* = 0.002), and only low dose (20 ng) of SDF-1 (α downregulated NOS2 mRNA levels (4.07 ± 0.03 vs. LPS 9.85 ± 1.62, *p* = 0.01).

## Abbreviations

DCM: dilated cardiomyopathy
EPC: endothelial progenitor cell
EPC-CFUs: endothelial progenitor cell-colony forming units
FMD: flow-mediated vasodilation
HF: heart failure
MSC: mesenchymal stem cell therapy
NAC: N-acetyl -cysteine
SDF-1α: stromal derived factor- 1 alpha
TNFα: tumor necrosis factor alpha.
