# Supplementary figures and images for: Mesenchymal Stem Cell Secretion of SDF-1α Modulates Endothelial Function in Dilated Cardiomyopathy
Source: Front Physiol. 2019 Sep 24;10:1182. doi: 10.3389/fphys.2019.01182 (PMC6769040; doi:10.3389/fphys.2019.01182)

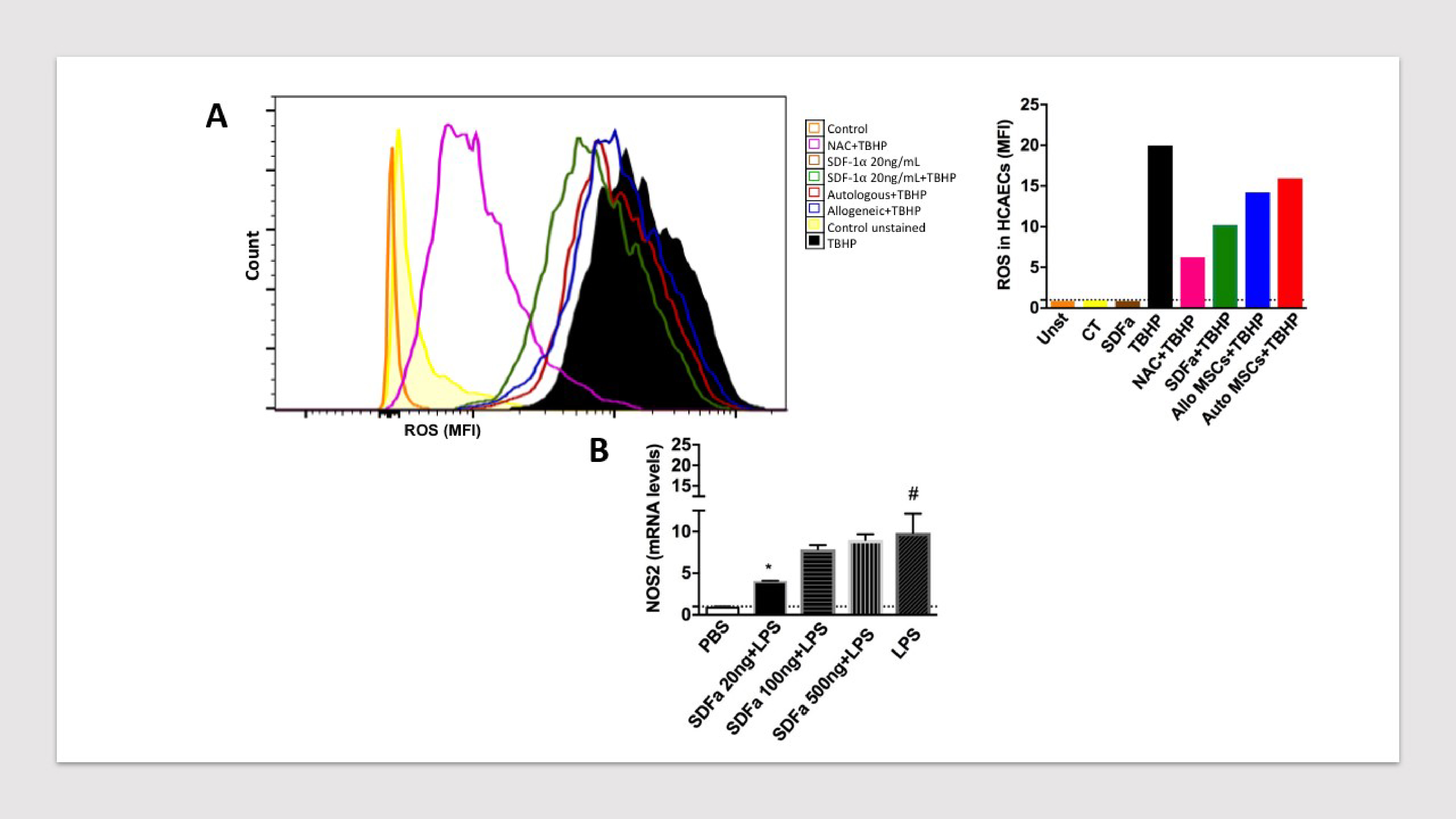

Supplement: FIGURE S1 — (A) HCAECs were incubated with cellROX and expression of ROS was measured via FACs. HCAECs pre-treated with SDF-1α allogeneic conditioned media and autologous conditioned medium lowered ROS production stimulated by TBHP in 50, 33, and 30%, respectively. (B) qPCR analysis of HCAECs stimulated with lipopolysaccharide (LPS) showed significantly upregulated NOS2 mRNA levels (9.85 ± 1.62 vs. PBS control 0.99 ± 0.01, p = 0.002), and only low dose (20 ng) of SDF-1 (α downregulated NOS2 mRNA levels (4.07 ± 0.03 vs. LPS 9.85 ± 1.62, p = 0.01). [file Image_1.JPEG]
